# Genetically predicted small dense low-density lipoprotein cholesterol and ischemic stroke subtype: multivariable Mendelian randomization study

**DOI:** 10.3389/fendo.2024.1404234

**Published:** 2024-07-29

**Authors:** Xiao Yu, Guangxun Shen, Yan Zhang, Cancan Cui, Yining Zha, Pingan Li, Lihong Li, Xu Wang, Guangxian Nan

**Affiliations:** ^1^ China-Japan Union Hospital of Jilin University, Jilin University, Jilin, China; ^2^ Department of Epidemiology, Harvard T. H. Chan School of Public Health, Boston, MA, United States; ^3^ School of Public Health, Capital Medical University, Beijing, China

**Keywords:** low-density lipoprotein cholesterol (LDL-C) subfractions, small low-density lipoprotein cholesterol (S-LDL-C), ischemic stroke (IS), large artery stroke (LAS), Mendelian randomization, low-density lipoprotein cholesterol (LDL-C), medium low-density lipoprotein cholesterol (M-LDL-C)

## Abstract

**Purpose:**

Small dense low-density lipoprotein cholesterol (S-LDL-C) has been suggested as a particularly atherogenic factor for ischemic stroke (IS) in observational studies, but the causality regarding the etiological subtype remains unclear. This study aims to explore the causal effects of small dense low-density lipoprotein cholesterol (S-LDL-C), medium (M-LDL-C) and large (L-LDL-C) subfractions on the lifetime risk of ischemic stroke (IS) and main subtypes using two-sample Mendelian randomization (TSMR) design.

**Methods:**

We identified genetic instruments for S-LDL-C, M-LDL-C and L-LDL-C from a genome-wide association study of 115 082 UK Biobank participants. Summary-level data for genetic association of any ischemic stroke (AIS), large artery stroke (LAS), small vessel stroke (SVS) and cardioembolic stroke (CES) were obtained from MEGASTROKE consortium. Accounting for the pleiotropic effects of triglycerides (TG) and high-density lipoprotein cholesterol (HDL-C), we conducted multivariable TSMR analysis.

**Results:**

In univariable TSMR, we found a causal association between genetically predicted S-LDL-C and LAS (IVW-FE: odds ratio (OR) = 1.481, 95% confidence interval (CI): 1.117–1.963, P = 0.006, q = 0.076) but not AIS, SVS or CES. No causal effects were observed for M-LDL-C or L-LDL-C in terms of AIS and IS subtype. In multivariable analysis, the causal association between S-LDL-C and LAS remained significant (IVE-MRE: OR = 1.329, 95% CI: 1.106–1.597, P = 0.002).

**Conclusions:**

Findings supported a causal association between S-LDL-C and LAS. Further studies are warranted to elucidate the underlying mechanism and clinical benefit of targeting S-LDL-C.

## Introduction

Stroke is a major global health burden, causing 6.55 million deaths and 143 million disability-adjusted life years worldwide in 2019 (1–3). Ischemic stroke (IS), accounting for more than three-quarters of all stroke cases, is the most common type of stroke, with a global prevalence of 77.19 million and a mortality of 3.29 million in 2019 (1, 3). IS can be further divided into five subtypes according to the Trial of ORG 10172 in Acute Stroke Treatment (TOAST) classification system (4). The main subtypes of IS are large artery stroke (LAS), small vessel stroke (SVS) and cardioembolic stroke (CES), which comprise nearly 95% of IS cases (5). On the other hand, low-density lipoprotein cholesterol (LDL-C) is a well-established risk factor for IS (6–8). It is noteworthy that high LDL-C levels are associated with an increased risk of LAS but not related to CES according to previous studies (9, 10). The evidence between LDL-C level and risk of SVS is inconsistent, suggesting a heterogeneity in terms of LDL-C and IS subtype (9, 10).

LDL-C is generally classified into three subfractions: small (S-LDL-C), medium (M-LDL-C) and large (L-LDL-C) LDL-C measured by nuclear magnetic resonance (NMR) spectroscopy. Small LDL particles could be more atherogenic than larger LDL particles because they are more susceptible to oxidation and have greater affinity for proteoglycans in the arterial wall (11). However, evidence of LDL-C subfractions and IS is scarce and inconsistent. Previous studies have reported a positive association between S-LDL-C level and the risk of IS (12–14). On the contrary, another study showed no association between S-LDL-C level and IS after adjusting for traditional risk factors (15), which is possibly attributed to the confounding bias existing in observational designs. Moreover, there are few studies about LDL-C subfractions and the risks of IS subtypes thus far.

Mendelian randomization (MR) provides an effective approach to quantify the causal relationship between lipids and stroke (16). In MR, genetic variants that are strongly associated with the exposure are used as instrumental variables to infer the causal association between the exposure and the outcome. Compared with observational studies, MR studies can effectively eliminate the bias of unmeasured confounders and reverse causation. Previous MR studies have investigated the effect of LDL-C on IS and IS subtypes (8, 9). Univariable analysis of a two-sample MR (TSMR) study indicated a positive causation between LDL-C and any ischemic stroke (AIS) (8). Another MR analysis with European participants also found that LDL-C is causally associated with AIS, LAS and SVS but not with CES (9). However, the causation of LDL-C subfractions with the risk of IS and its main subtypes remains unclear. Therefore, we performed univariate and multivariate TSMR analyses to evaluate the causal relationship between LDL-C subfractions and the risk of IS and its main subtypes in the context of precision medicine.

## Methods

### Study design

We used publicly available summary-level data from genome-wide association studies (GWAS) to perform MR analysis. MR analysis relies on three key assumptions of instrumental variables (IVs). The first assumption is that IVs should be strongly associated with the exposure; the second assumption is that IVs should be independent of any confounders; and the third assumption is that IVs should only affect the outcome through the exposure and not through other pathways (17). The horizontal pleiotropy of IVs means that IVs are associated with exposure as well as other phenotypes. The horizontal pleiotropy of IVs violates the second or the third assumption of IVs and biases the MR results. Only IVs that meet all three assumptions can be considered valid. The validity of IVs has a major impact on the reliability of MR results.

### Data sources

We obtained summary-level instrumental data of LDL-C subfractions, high-density lipoprotein cholesterol (HDL-C) and triglycerides (TG) from a GWAS study based on plasma measurements from 115 082 UK Biobank participants of European ancestry (18). The measurements quantified 249 metabolic biomarkers, including LDL-C subfractions, HDL-C, TG and other small molecules using the Nightingale Health NMR biomarker platform (19).

We obtained summary-level GWAS data of any ischemic stroke (AIS) and IS subtypes, including LAS, SVS and CES, from a meta-analysis of 17 GWAS studies conducted by the MEGASTROKE consortium (20). This meta-analysis included 446 696 individuals of European ancestry, comprising 40 585 cases of any stroke (AS) and 406 111 controls. All cases of AS were diagnosed according to the World Health Organization definition of stroke, and AS cases were classified into AIS and intracerebral hemorrhage based on clinical and imaging criteria. AIS cases were further divided into subtypes according to the TOAST classification system. The sources and details of the data used in this current study are shown in **Additional File 1: Supplementary Table S1**.

### Instrumental variable selection

We used the following selection criteria to select IVs in the univariable MR analysis: (1) We extracted single-nucleotide polymorphisms (SNPs) significantly associated with total LDL-C, S-LDL-C, M-LDL-C or L-LDL-C (P < 5×10^-8^) as potential IVs for each exposure. (2) To avoid linkage disequilibrium (LD) among IVs, we calculated the LD parameter (r^2^) between SNPs based on the reference panel consisting of 1000 Genomes Project European sample data. We assessed the independence of SNPs using stringent criteria (r^2^<0.001; clumping window, 10000 kb). We excluded SNPs that were not available in the reference panel data. (3) To restrict the potential horizontal pleiotropy of IVs, we excluded SNPs that were present in two or more IV groups of exposures. (4) We extracted information of each SNP corresponding with the AIS, LAS, SVS and CES GWAS data. We excluded SNPs that were unavailable in the outcome data and did not have any proxy SNP with r^2^>0.8. We also removed SNPs with a minor allele frequency (MAF) less than 0.3. (5) For palindromic SNPs, we used allele frequency information to infer the correct forward strand allele. We removed palindromic SNPs with MAF above 0.42 that could not be inferred as the forward strand allele and SNPs with inconsistent alleles in outcome data and exposure data. (6) We conducted a Steiger filtering test on each remaining SNP. The test calculated the variance of the exposure and the outcome explained by the SNP. We removed SNPs that explained less variance of the exposure than the variance of the outcome. The numbers of IVs in each group of exposures and outcomes in univariable MR analysis are shown in **Additional File 1: Supplementary Table S2**.

We selected independent SNPs (r^2^<0.001, kb=10000) that were significantly associated with at least one exposure and available or had proxy SNPs (r^2^>0.8) available in all exposure data and the outcome data as IVs in the multivariable MR analysis.

### Statistical analysis

We used the inverse variance weighted fixed effects (IVW-FE) method as the main analysis of univariable MR. IVW-FE combines causal estimates of each SNP with the weight of reciprocal of outcome variance. The causal estimate of a single SNP is calculated by using the Wald ratio method, and the standard error of the causal estimate is calculated by the delta method (21). The IVW-FE method requires each IV to be valid, which means that all IVs in MR analyses do not have horizontal pleiotropy. The IVW-FE method also requires the NO Measurement Error (NOME) assumption, which means variances of association estimates between SNPs and exposures are negligible (22). When these conditions are satisfied, the IVW-FE method can provide an unbiased result with the highest power (23, 24).

To examine the robustness of the IVW-FE result, we also used five methods including inverse variance weighted multiplicative random effects (IVW-MRE), weighted median, MR−Egger, MR-robust adjusted profile score (MR-RAPS) and MR-constrained maximum likelihood and model averaging and Bayesian information criterion (MR-cML-MA-BIC) method as complementary analyses of univariable MR. IVW-MRE method assumes that SNPs only have balanced uncorrelated horizontal pleiotropy, which means the total effect of uncorrelated horizontal pleiotropy of all SNPs is zero (25). The weighted median method allows a fraction of SNPs to violate the second or the third IV assumption but requires more than half of the SNPs to be valid (26). The MR−Egger method can generate reliable estimates even if all SNPs violate the third IV assumption but requires the NOME assumption and Instrument Strength Independent of Direct Effect (InSIDE) assumption to be satisfied (27). The relaxation of SNP restrictions lowers the statistical power of the MR−Egger method at the same time. The MR-RAPS approach can provide consistent estimates even if all SNPs violated the third IV assumption (26). The MR-RAPS approach also allows many weak IVs to exist in analyses (23, 28). The MR-cML-MA-BIC method allows some SNPs to violate the second and third IV assumptions and requires only a plurality of SNPs to be valid (26). Moreover, compared with the weighted median approach, the MR-cML-MA-BIC method has higher power and a lower false positive error rate (26). To address multiple hypothesis testing, we conducted false discovery rate (FDR) correction by calculating the q value (29). We considered univariable MR results with P values below 0.05 and q values below 0.1 to have strong evidence of causal association; results with P values below 0.05 but q values above 0.1 to have suggestive causations between exposures and outcomes; and results with P values above 0.05 to have no correlation between the exposure and the outcome.

To test the robustness of univariable MR results, we performed several sensitivity analyses as follows: (1) We assessed the strength of the IV using the F-statistic, calculated as F = (N-2) × R^2^/(1-R^2^), where N is the sample size of the exposure GWAS data and R^2^ is the proportion of variance explained by the SNP (30, 31). R^2^ was derived from R^2^ = [2×(1-EAF)×EAF×β^2^]/SD^2^, where EAF is the effect allele frequency, β is the estimate of the association between the SNP and the exposure, SD is the standard deviation of the exposure sample and was obtained from SD = SE×N^1/2^, where SE is the standard error of β. An F-statistic ≤ 10 indicated a possibility of weak instrument that could bias the MR result (32). (2) We quantified the degree of violation of the NOME assumption in the MR−Egger approach using I_GX_^2^ statistics. A severe violation of NOME could bias the MR-Egger estimate toward zero and underestimate the causal effect (22). I_GX_^2^<0.9 suggested a high degree of violation that could influence the MR−Egger result (33). (3) We measured the heterogeneity of IVs in the IVW-FE method using Cochran’s Q and I^2^ statistics. A P value below 0.05 in Cochran’s Q test indicated significant heterogeneity among SNPs. I^2^ represented the proportion of variance in the causal estimate due to heterogeneity. I^2^≥40% suggested substantial heterogeneity. (4) We detected horizontal pleiotropy in IVs using the MR−Egger intercept test and MR-Pleiotropy RESidual Sum and Outlier (MR-PRESSO) test. A significant deviation of the MR−Egger intercept from zero (P<0.05) implied unbalanced uncorrelated horizontal pleiotropy in IVs. The MR-PRESSO global test evaluated the overall uncorrelated horizontal pleiotropy in IVs. A P value below 0.05 in the MR-PRESSO global test indicated horizontal pleiotropy in IVs. The MR-PRESSO outlier test identified potential outliers from the MR analysis. A P value below 0.05 in the MR−PRESSO outlier test suggested that the SNP was an outlier.

We examined whether the SNPs used in univariable MR analysis were also associated with TG or HDL-C, which were reported to be risk or protective factors of AIS, LAS and SVS (9) and correlated with S-LDL-C level (34, 35). We retrieved previously published GWAS data of each SNP from the PhenoScanner V2 database (36). Among the 26 SNPs of S-LDL-C, 2 SNPs (rs4846914 and rs4810479) were associated with HDL-C, and 4 SNPs (rs4846914, rs687420, rs6167975 and rs4810479) were associated with TG. Among the 27 SNPs of M-LDL-C, 1 SNP (rs2144300) was associated with HDL-C, and 2 SNPs (rs2144300 and rs6073958) were associated with TG. Among the 58 SNPs of L-LDL-C, 7 SNPs (rs1461729, rs11789603, rs2792735, rs261290, rs633695, rs72836561 and rs1800961) were associated with HDL-C, and 7 SNPs (rs2642438, rs59950280, rs4722551, rs17411113, rs525028, rs633695 and rs7254892) were associated with TG. To further control for potential horizontal pleiotropy, we performed multivariable MR analysis for the pairs of exposures and outcome that showed strong or suggestive evidence of causality in univariable MR analysis, including both TG and HDL-C as exposures. A P value below 0.05 in multivariable MR analysis indicated a strong causal association.

The effect estimates of exposures on outcomes were presented as odds ratios (ORs) with their 95% confidence intervals (CIs) per 1-standard-deviation-higher of exposures.

All statistical analyses were conducted using R (version 4.2.3) packages, including TwoSampleMR, MRPRESSO, mr.raps, MRcML, metafor and qvalue.

## Results

### Univariable MR

**Figure 1:**
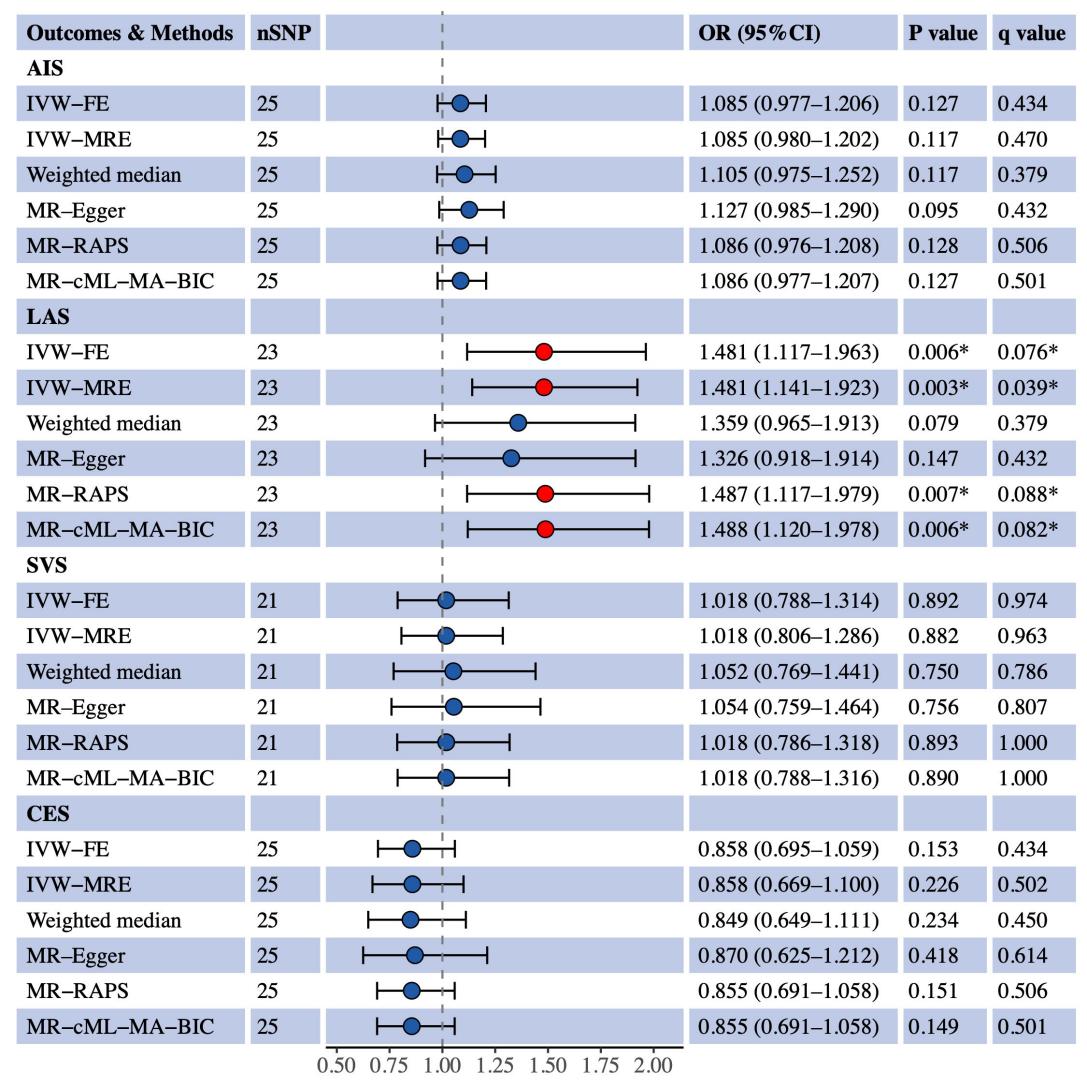
Univariable MR estimates for the causal effect of S-LDL-C on AIS, LAS, SVS and CES. SNP, single-nucleotide polymorphism; OR, odds ratio; CI, confidence interval; AIS, any ischemic stroke; IVW-FE, inverse variance weighted fixed effects; IVE-MRE, inverse variance weighted multiplicative random effects; MR-RAPS, MR-robust adjusted profile score; MR-cML-MA-BIC, MR-constrained maximum likelihood and model averaging and Bayesian information criterion; LAS, large artery stroke; SVS, small vessel stroke; CES, cardioembolic stroke. *Indicating P value<0.05 or q value<0.1.

**Figure 2:**
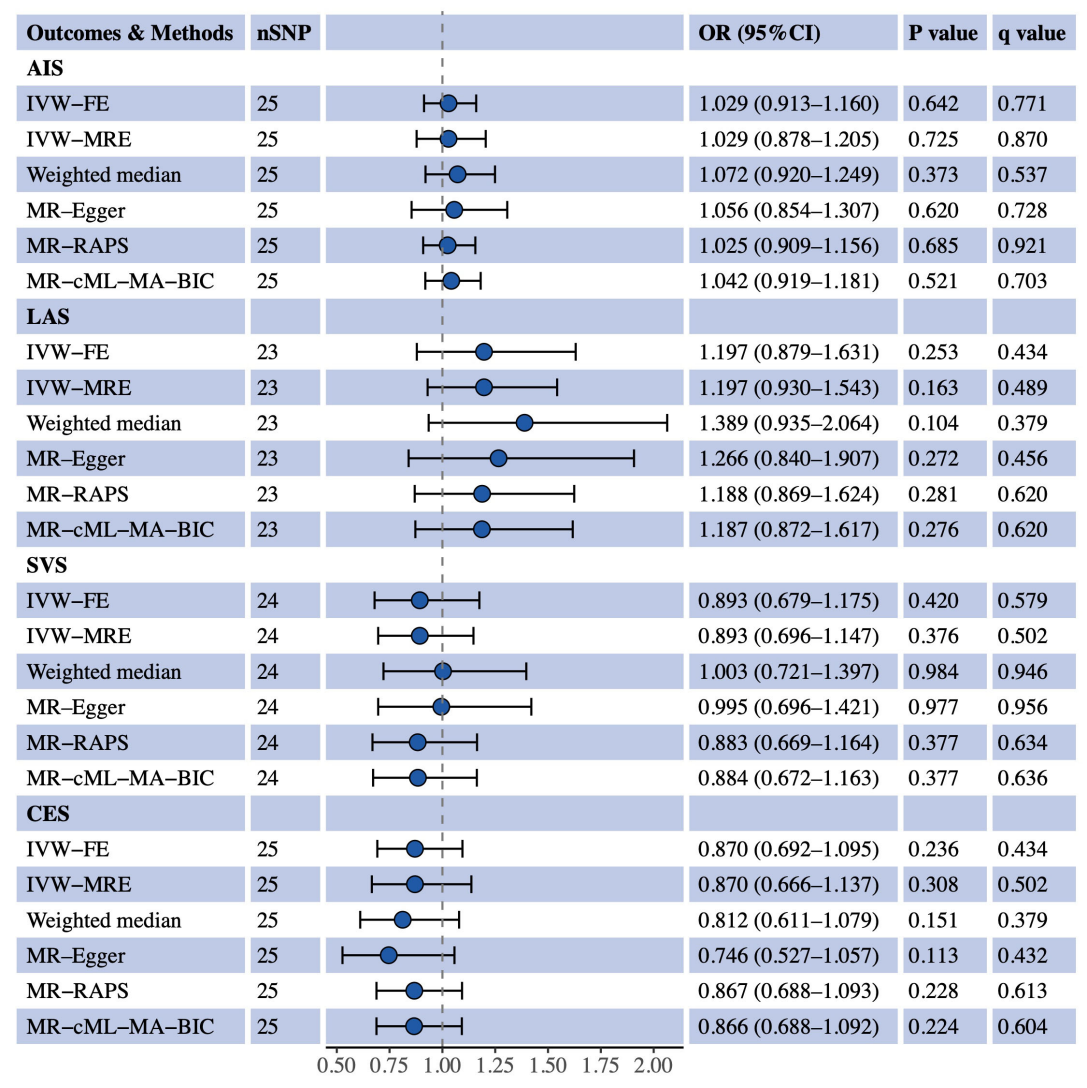
Univariable MR estimates for the causal effect of M-LDL-C on AIS, LAS, SVS and CES. SNP, single-nucleotide polymorphism; OR, odds ratio; CI, confidence interval; AIS, any ischemic stroke; IVW-FE, inverse variance weighted fixed effects; IVE-MRE, inverse variance weighted multiplicative random effects; MR-RAPS, MR-robust adjusted profile score; MR-cML-MA-BIC, MR-constrained maximum likelihood and model averaging and Bayesian information criterion; LAS, large artery stroke; SVS, small vessel stroke; CES, cardioembolic stroke.

**Figure 3:**
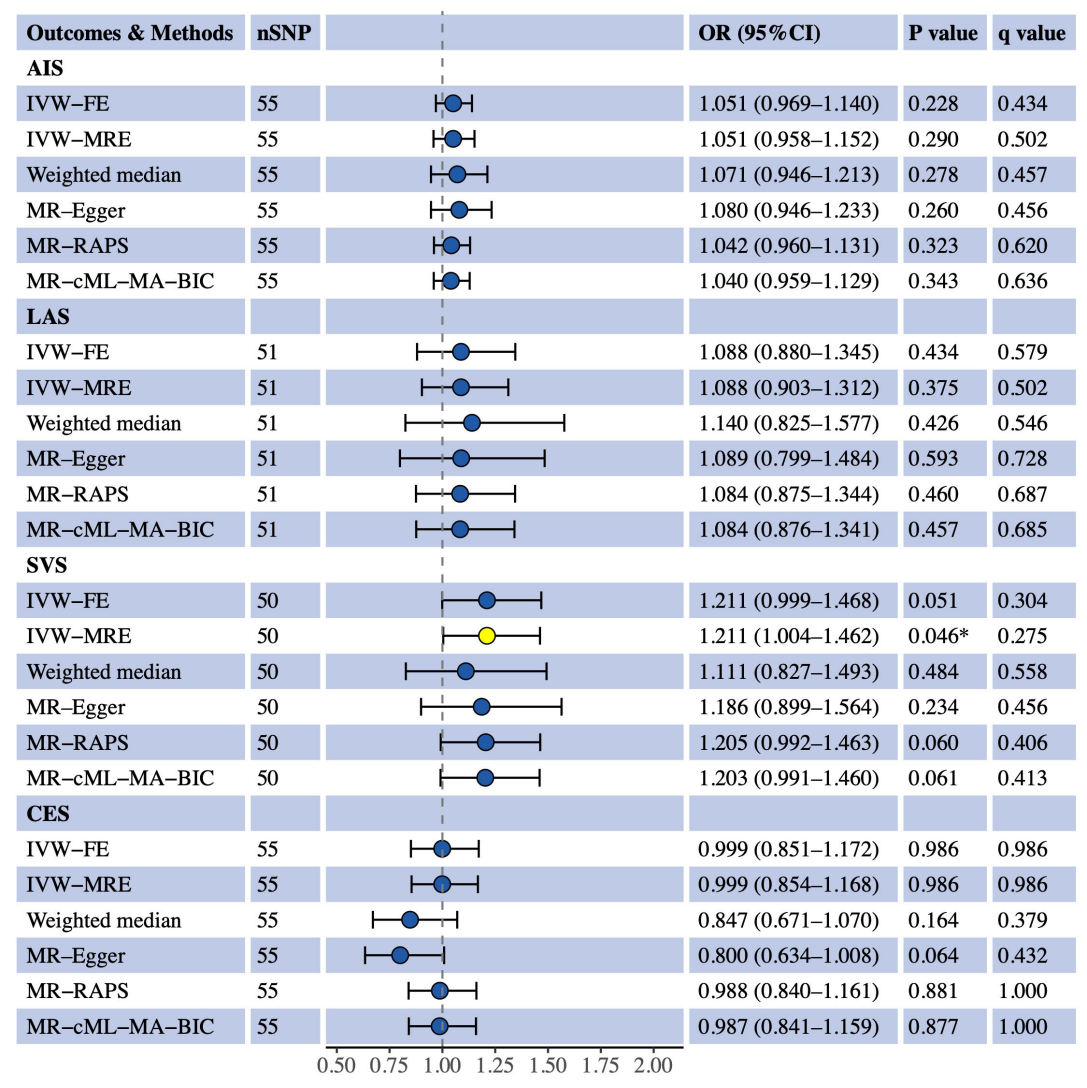
Univariable MR estimates for the causal effect of L-LDL-C on AIS, LAS, SVS and CES. SNP, single-nucleotide polymorphism; OR, odds ratio; CI, confidence interval; AIS, any ischemic stroke; IVW-FE, inverse variance weighted fixed effects; IVE-MRE, inverse variance weighted multiplicative random effects; MR-RAPS, MR-robust adjusted profile score; MR-cML-MA-BIC, MR-constrained maximum likelihood and model averaging and Bayesian information criterion; LAS, large artery stroke; SVS, small vessel stroke; CES, cardioembolic stroke. *sIndicating P value<0.05.

We used univariable MR to estimate the causal effects of S-LDL-C, M-LDL-C and L-LDL-C on AIS and three IS subtypes (LAS, SVS and CES). **Figure 1** shows the MR results for S-LDL-C. The IVW-FE method indicated a positive causal association between S-LDL-C and LAS (OR = 1.481, 95% CI: 1.117–1.963, P = 0.006, q = 0.076). Three of the five complementary methods (IVW-MRE, MR-RAPS and MR-cML-MA-BIC) also supported the finding. No evidence of causal effects of S-LDL-C on AIS, SVS and CES was detected by any method. **Figures 2**, **3** show the MR results for M-LDL-C and L-LDL-C, respectively. None of the methods found a causal effect of M-LDL-C on AIS or any AIS subtype. The IVW-MRE method indicated a suggestively positive causal effect of L-LDL-C on SVS (OR = 1.211, 95% CI: 1.004–1.462, P = 0.046, q = 0.275), which was not confirmed by other methods. In addition, we validated that total LDL-C was causally associated with AIS and LAS consistent with prior literatures (**Additional File 1: Supplementary Figure S1**).

### Sensitivity analysis

We assessed the heterogeneity and pleiotropy of the IVs used in univariable MR analysis. **Table 1** shows the results of Cochran’s Q test and I^2^ statistics for heterogeneity. The only significant heterogeneity was found for S-LDL-C and AIS (P = 0.013, I^2^ = 42.63%). **Table 2** shows the results of the MR−Egger intercept test and MR-PRESSO global test for pleiotropy. The results of the two tests were inconsistent for L-LDL-C and CES (MR−Egger, P = 0.012; MR-PRESSO, P = 0.479), and L-LDL-C and AIS (MR−Egger, P = 0.575; MR-PRESSO, P = 0.027). There was insufficient evidence for horizontal pleiotropy in these pairs. No evidence of horizontal pleiotropy was found for the other pairs by either test.

**Table 1:** Results of heterogeneity test between LDL-C subfractions and AIS and main subtypes.

Exposures	Outcomes	Q	Q-P value	I^2^
S-LDL-C	AIS	22.751	0.535	0.00%
S-LDL-C	LAS	18.909	0.651	0.00%
S-LDL-C	SVS	16.743	0.670	0.00%
S-LDL-C	CES	33.420	0.096	28.19%
M-LDL-C	AIS	41.836	0.013	42.63%
M-LDL-C	LAS	14.778	0.872	0.00%
M-LDL-C	SVS	19.149	0.693	0.00%
M-LDL-C	CES	32.424	0.117	25.98%
L-LDL-C	AIS	70.007	0.070	22.86%
L-LDL-C	LAS	38.867	0.873	0.00%
L-LDL-C	SVS	46.906	0.558	0.00%
L-LDL-C	CES	51.685	0.564	0.00%

S-LDL-C, small low-density lipoprotein cholesterol; M-LDL-C, medium low-density lipoprotein cholesterol; L-LDL-C, large low-density lipoprotein cholesterol; AIS, any ischemic stroke; LAS, large artery stroke; SVS, small vessel stroke; CES, cardioembolic stroke.

**Table 2:** Results of pleiotropy test between LDL-C subfractions and AIS and IS main subtypes.

Exposures	Outcomes	Intercept (MR−Egger)	Standard error (MR−Egger)	P value (MR−Egger)	P value (MR-PRESSO global test)
S-LDL-C	AIS	-0.003	0.003	0.385	0.594
S-LDL-C	LAS	0.008	0.009	0.367	0.671
S-LDL-C	SVS	-0.003	0.008	0.742	0.752
S-LDL-C	CES	-0.001	0.008	0.897	0.167
M-LDL-C	AIS	-0.002	0.005	0.715	0.057
M-LDL-C	LAS	-0.004	0.009	0.689	0.860
M-LDL-C	SVS	-0.007	0.008	0.366	0.679
M-LDL-C	CES	0.010	0.007	0.200	0.207
L-LDL-C	AIS	-0.002	0.003	0.575	0.027
L-LDL-C	LAS	0.000	0.007	0.997	0.871
L-LDL-C	SVS	0.001	0.007	0.835	0.572
L-LDL-C	CES	0.014	0.005	0.012	0.479

MR-PRESSO, MR-Pleiotropy RESidual Sum and Outlier; S-LDL-C, small low-density lipoprotein cholesterol; M-LDL-C, medium low-density lipoprotein cholesterol; L-LDL-C, large low-density lipoprotein cholesterol; AIS, any ischemic stroke; LAS, large artery stroke; SVS, small vessel stroke; CES, cardioembolic stroke.

We also calculated the F-statistics of each IV to check for weak instruments (**Additional File 1: Supplementary Tables S3**–**S5**). The number of IVs with F-statistics below 10 is shown in **Additional File 1: Supplementary Table S6**. Although some potential weak instruments were detected, the estimates of ORs in MR-RAPS were consistent with those in IVW-FE for all pairs of exposures and outcomes, supporting the robustness of our univariable MR results. The I_GX_^2^ statistics for all pairs were above 0.9 (**Additional File 1: Supplementary Table S7**), suggesting that the violation of the NOME assumption was mild when using the MR−Egger method. No evidence suggested that the NOME violation had a substantial impact on the MR−Egger results. The leave-one-out analysis suggested that the observed findings between S-LDL-C and LAS were robust S-LDL-C (**Additional File 1: Supplementary Figure S3**).

### Multivariable MR

**Figure 4:**
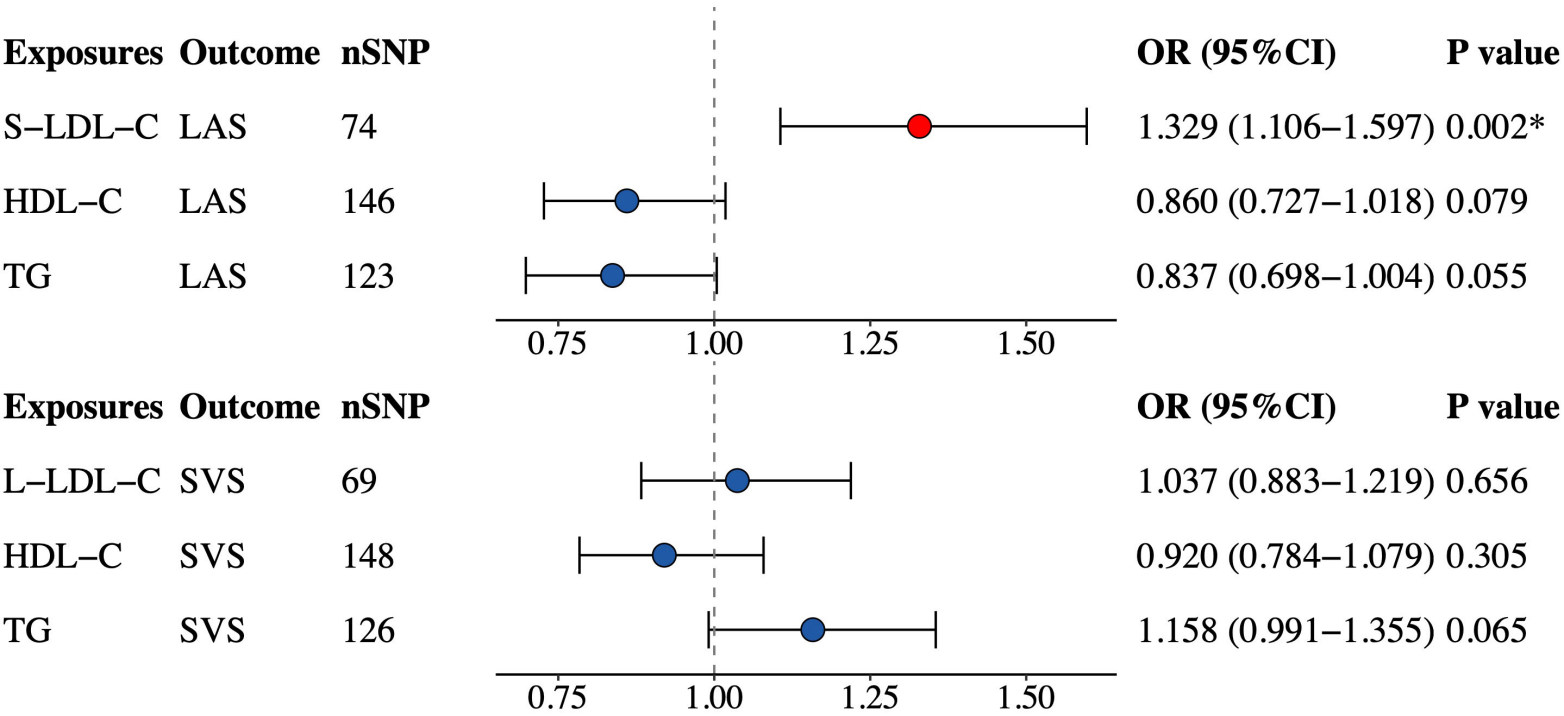
Multivariable MR estimates for the causal effect of S-LDL-C on LAS and L-LDL-C on SVS. SNP, single-nucleotide polymorphism; OR, odds ratio; CI, confidence interval; S-LDL-C, small low-density lipoprotein cholesterol; LAS, large artery stroke; HDL-C, high-density lipoprotein cholesterol; TG, triglycerides; L-LDL-C, large low-density lipoprotein cholesterol; SVS, small vessel stroke. *Indicating P value<0.05.

To account for possible confounding effects, we included TG and HDL-C in the multivariable TSMR analysis of S-LDL-C and LAS plus L-LDL-C and SVS based on significant findings in univariable analyses. The multivariable MR analysis (**Figure 4**) confirmed a positive causal link between S-LDL-C and LAS (OR = 1.329, 95% CI: 1.106–1.597, P = 0.002). However, the association between L-LDL-C and SVS was not statistically significant in multivariable MR analysis (OR = 1.037, 95% CI: 0.883–1.219, P = 0.656). Considering the effects of BMI and glucose on IS, we additionally adjusted BMI and fasting glucose in the multivariable MR analysis, and the effect size between S-LDL-C and LAS was moderately attenuated (OR = 1.217, 95% CI: 1.086–1.403, P = 0.013).

## Discussion

Our study revealed a positive causal association between S-LDL-C and LAS but no other IS subtype. We could not claim any causal association of M-LDL-C or L-LDL-C with AIS or IS subtypes. These findings were robust to multiple sensitivity analyses, providing a potential target for the precise risk prevention of IS in the context of lipid management.

Previous observational studies have examined the links between LDL-C subfractions and AIS with inconsistent results (12, 13). The Copenhagen General Population Study found that higher S-LDL-C levels were robustly associated with an increased risk of ischemic stroke (14). A cohort study in Japanese participants also indicated that the risk of AIS increased with higher serum S-LDL-C levels (37). However, another prospective study showed no significant difference in the risk of AIS between participants with the highest and the lowest quartiles of baseline S-LDL-C levels (15). Our study used TSMR analysis to investigate the causal association between S-LDL-C and IS. Contrary to most previous studies, our study did not find any evidence of an overall association between S-LDL-C and AIS, suggesting the heterogeneity in terms of the associations between S-LDL-C and IS subtypes. Findings showed that S-LDL-C was a robust and causal risk factor for LAS. S-LDL-C may affect LAS risk through its atherogenic effect (38). S-LDL-C can influence atherosclerosis development directly and indirectly. The direct mechanism involves the physical and chemical properties of small low-density lipoprotein (S-LDL) particles. S-LDL particles have smaller diameters that allow them to penetrate the tunica intima, and S-LDL particles can persist longer in the bloodstream and enhance the atherogenic effect of cholesterol (38). The indirect mechanism involves the mediation of metabolic diseases. S-LDL-C may increase atherosclerosis risk by promoting these metabolic diseases such as diabetes (38).

In the univariable MR analysis of S-LDL-C and LAS, two complementary methods did not reach statistical significance, but both indicating a risk trend, consistent with other MR methods. The null results may be due to the low statistical power of these methods. The heterogeneity tests, pleiotropy tests and three complementary MR analyses all supported the validity of the IVW-FE result for S-LDL-C and LAS. Integrating the results of multivariable MR analysis, there is strong evidence to claim that S-LDL-C is a risk factor for LAS, independent of HDL-C and TG levels. The causal association between L-LDL-C and SVS was not confirmed in multivariable MR analysis, although IVW-MRE indicated a suggestive association between L-LDL-C and SVS.

Some of the IVs in the univariable MR analyses had low F-statistics, indicating potential weak instruments. In two-sample MR, weak instruments can only bias the odds ratio estimates toward 1, which does not affect the I error rate substantially, so the positive MR results are still reliable. Moreover, the extent of the weak instrument bias depends on the degree of overlap between the two samples (39). In this study, we used UK Biobank data for LDL-C subfractions and MEGASTROKE data for IS, which had no population overlap between exposure and outcome samples. Furthermore, we performed MR-RAPS analysis, which can provide robust causal estimates in the presence of many weak instruments, and the MR-RAPS results were consistent with the IVW-FE estimates. Therefore, we have insufficient evidence that the weak instrument bias had a noticeable impact on univariable MR results.

Several limitations should be acknowledged. First, our study was based on GWAS data from European populations, and the finding may not be generalizable to other ethnic groups. Second, some of the IVs were weak instruments, which may increase the false negative rate of univariable MR analyses, so the negative results should be interpreted cautiously. Third, the MR estimates may differ from the real-world effects due to the fact that MR reflects the effect of lifetime exposure. Future studies are needed to explore the causal association between the time-varying LDL-C subfractions and the risk of IS under a context of key time-point exposure (40) and clarify the interaction between LDL-C and other causal lipid markers (16).

In summary, the univariable and multivariable TSMR analyses indicated a positive causal association between S-LDL-C and LAS. Future studies should elucidate the underlying mechanism and the clinical benefit of treating S-LDL-C as a risk management target for the early prevention of IS and LAS.

## Data availability statement

The original contributions presented in the study are included in the article/**Supplementary Material**. Further inquiries can be directed to the corresponding authors.

## Ethics statement

Ethical approval was not required for the study involving humans in accordance with the local legislation and institutional requirements. Written informed consent to participate in this study was not required from the participants or the participants’ legal guardians/next of kin in accordance with the national legislation and the institutional requirements.

## Author contributions

XY: Writing – original draft. GS: Writing – original draft. YZ: Writing – original draft. CC: Writing – review & editing. YNZ: Writing – review & editing. PL: Writing – original draft. LL: Writing – review & editing. XW: Writing – review & editing. GN: Writing – original draft, Writing – review & editing.

## Glossary

**Table:**

LDL-C	Low-density lipoprotein cholesterol
IS	ischemic stroke
S-LDL-C	small low-density lipoprotein cholesterol
M-LDL-C	medium low-density lipoprotein cholesterol
L-LDL-C	large low-density lipoprotein cholesterol
TSMR	two-sample Mendelian randomization
AIS	any ischemic stroke
LAS	large artery stroke
SVS	small vessel stroke
CES	cardioembolic stroke
IVW-FE	inverse variance weighted fixed effects
TG	triglycerides
HDL-C	high-density lipoprotein cholesterol
OR	odds ratio
CI	confidence interval
TOAST	Trial of ORG 10172 in Acute Stroke Treatment
NMR	nuclear magnetic resonance
MR	Mendelian randomization
GWAS	genome-wide association studies
IVs	instrumental variables
AS	any stroke
SNPs	single-nucleotide polymorphisms
LD	linkage disequilibrium
MAF	minor allele frequency
NOME	NO Measurement Error
IVW-MRE	inverse variance weighted multiplicative random effects
MR-RAPS	MR-robust adjusted profile score
MR-cML-MA-BIC	MR-constrained maximum likelihood and model averaging and Bayesian information criterion
InSIDE	Instrument Strength Independent of Direct Effect
FDR	false discovery rate
MR-PRESSO	MR-Pleiotropy RESidual Sum and Outlier
HR	hazard ratio
