# Supplementary material for: Genetically predicted small dense low-density lipoprotein cholesterol and ischemic stroke subtype: multivariable Mendelian randomization study
Source: Front Endocrinol (Lausanne). 2024 Jul 29;15:1404234. doi: 10.3389/fendo.2024.1404234 (PMC11317295; doi:10.3389/fendo.2024.1404234)

**Additional file 1**

**Table S1.** Sources and details of data for the analysis.

| **Phenotype** | **Data source** | **PMID** | **Sample size** | **Cases** | **Controls** | **SNPs** |
| --- | --- | --- | --- | --- | --- | --- |
| **Exposures** |  |  |  |  |  |  |
| LDL-C | UK Biobank | 35213538 | 115,082 | - | - | 12,321,875 |
| S-LDL-C | UK Biobank | 35213538 | 115,082 | - | - | 12,321,875 |
| M-LDL-C | UK Biobank | 35213538 | 115,082 | - | - | 12,321,875 |
| L-LDL-C | UK Biobank | 35213538 | 115,082 | - | - | 12,321,875 |
| HDL-C | UK Biobank | 35213538 | 115,082 | - | - | 12,321,875 |
| TG | UK Biobank | 35213538 | 115,082 | - | - | 12,321,875 |
| **Outcomes** |  |  |  |  |  |  |
| AIS | MEGASTROKE | 29531354 | 440,328 | 34,217 | 406,111 | 8,296,492 |
| LAS | MEGASTROKE | 29531354 | 150,765 | 4,373 | 146,392 | 8,418,349 |
| SVS | MEGASTROKE | 29531354 | 198,048 | 5,386 | 192,662 | 8,280,845 |
| CES | MEGASTROKE | 29531354 | 211,763 | 7,193 | 204,570 | 8,271,294 |

Abbreviations: SNPs, single-nucleotide polymorphisms; S-LDL-C, small low-density lipoprotein cholesterol; M-LDL-C, medium low-density lipoprotein cholesterol; L-LDL-C, large low-density lipoprotein cholesterol; HDL-C, high-density lipoprotein cholesterol; TG, triglycerides; AIS, any ischemic stroke; LAS, large artery stroke; SVS, small vessel stroke; CES, cardioembolic stroke.

**Table S2.** Numbers of IVs in each group of exposures and outcomes in univariable MR.

|  | **LDL-C** | **S-LDL-C** | **M-LDL-C** | **L-LDL-C** |
| --- | --- | --- | --- | --- |
| **AIS** | 105 | 25 | 25 | 55 |
| **LAS** | 98 | 23 | 23 | 51 |
| **SVS** | 98 | 21 | 24 | 50 |
| **CES** | 104 | 25 | 25 | 55 |

Abbreviations: S-LDL-C, small low-density lipoprotein cholesterol; M-LDL-C, medium low-density lipoprotein cholesterol; L-LDL-C, large low-density lipoprotein cholesterol; AIS, any ischemic stroke; LAS, large artery stroke; SVS, small vessel stroke; CES, cardioembolic stroke.

**Table S3.** SNPs information and F statistics of S-LDL-C.

| **RSID** | **EAF** | **β** | **Standard error of β** | **Sample size** | **R^2^** | **F** |
| --- | --- | --- | --- | --- | --- | --- |
| rs4846914 | 0.605 | -0.025 | 4.229×10^-3^ | 115082 | 1.433×10^-4^ | 16.493 |
| rs7580634 | 0.424 | -0.020 | 4.193×10^-3^ | 115082 | 9.750×10^-5^ | 11.221 |
| rs13059141 | 0.113 | -0.032 | 6.515×10^-3^ | 115082 | 4.321×10^-5^ | 4.973 |
| rs1784166 | 0.763 | 0.021 | 4.873×10^-3^ | 115082 | 6.062×10^-5^ | 6.977 |
| rs6874202 | 0.634 | 0.040 | 4.283×10^-3^ | 115082 | 3.539×10^-4^ | 40.740 |
| rs1562125 | 0.193 | 0.023 | 5.237×10^-3^ | 115082 | 5.020×10^-5^ | 5.778 |
| rs115740542 | 0.074 | -0.061 | 7.953×10^-3^ | 115082 | 6.896×10^-5^ | 7.936 |
| rs4252093 | 0.293 | 0.019 | 4.552×10^-3^ | 115082 | 6.493×10^-5^ | 7.472 |
| rs3777411 | 0.151 | -0.046 | 5.778×10^-3^ | 115082 | 1.406×10^-4^ | 16.180 |
| rs7746472 | 0.179 | -0.024 | 5.416×10^-3^ | 115082 | 5.149×10^-5^ | 5.926 |
| rs56217912 | 0.605 | 0.020 | 4.743×10^-3^ | 115082 | 6.075×10^-5^ | 6.991 |
| rs2107449 | 0.257 | 0.020 | 4.266×10^-3^ | 115082 | 9.295×10^-5^ | 10.698 |
| rs4732167 | 0.380 | -0.047 | 1.049×10^-2^ | 115082 | 1.373×10^-5^ | 1.580 |
| rs2737263 | 0.960 | -0.022 | 4.602×10^-3^ | 115082 | 7.809×10^-5^ | 8.988 |
| rs11057601 | 0.280 | -0.020 | 4.212×10^-3^ | 115082 | 9.253×10^-5^ | 10.649 |
| rs6602909 | 0.591 | 0.024 | 4.404×10^-3^ | 115082 | 1.138×10^-4^ | 13.101 |
| rs9567639 | 0.329 | -0.026 | 5.036×10^-3^ | 115082 | 7.628×10^-5^ | 8.778 |
| rs35475746 | 0.214 | 0.032 | 7.571×10^-3^ | 115082 | 2.403×10^-5^ | 2.766 |
| rs35334131 | 0.084 | -0.034 | 7.588×10^-3^ | 115082 | 2.735×10^-5^ | 3.148 |
| rs143351043 | 0.083 | 0.095 | 2.062×10^-2^ | 115082 | 4.534×10^-6^ | 0.522 |
| rs79983326 | 0.012 | -0.030 | 6.430×10^-3^ | 115082 | 4.040×10^-5^ | 4.649 |
| rs2738447 | 0.118 | 0.033 | 4.206×10^-3^ | 115082 | 2.549×10^-4^ | 29.337 |
| rs61679753 | 0.593 | -0.458 | 1.198×10^-2^ | 115082 | 7.541×10^-4^ | 86.853 |
| rs4810479 | 0.031 | -0.038 | 4.775×10^-3^ | 115082 | 2.109×10^-4^ | 24.279 |
| rs6016590 | 0.750 | 0.018 | 4.189×10^-3^ | 115082 | 7.786×10^-5^ | 8.961 |

Abbreviations: EAF, effect allele frequency.

**Table S4.** SNPs information and F statistics of M-LDL-C.

| **RSID** | **EAF** | **β** | **Standard error of β** | **Sample size** | **R^2^** | **F** |
| --- | --- | --- | --- | --- | --- | --- |
| rs7529815 | 0.351 | -0.018 | 4.343×10^-3^ | 115082 | 6.947×10^-5^ | 7.995 |
| rs5000342 | 0.694 | -0.020 | 4.518×10^-3^ | 115082 | 7.114×10^-5^ | 8.187 |
| rs2144300 | 0.605 | -0.021 | 4.226×10^-3^ | 115082 | 1.018×10^-4^ | 11.716 |
| rs13409360 | 0.397 | -0.020 | 4.254×10^-3^ | 115082 | 9.598×10^-5^ | 11.046 |
| rs164641 | 0.049 | -0.046 | 9.650×10^-3^ | 115082 | 1.854×10^-5^ | 2.133 |
| rs6814828 | 0.149 | 0.026 | 5.841×10^-3^ | 115082 | 4.220×10^-5^ | 4.857 |
| rs1263493 | 0.764 | 0.022 | 4.882×10^-3^ | 115082 | 6.45×10^-5^ | 7.424 |
| rs1016988 | 0.191 | -0.024 | 5.270×10^-3^ | 115082 | 5.454×10^-5^ | 6.277 |
| rs80145669 | 0.029 | -0.052 | 1.233×10^-2^ | 115082 | 8.599×10^-6^ | 0.990 |
| rs4708870 | 0.059 | -0.047 | 8.796×10^-3^ | 115082 | 2.791×10^-5^ | 3.212 |
| rs147290678 | 0.179 | -0.025 | 5.398×10^-3^ | 115082 | 5.281×10^-5^ | 6.078 |
| rs1989985 | 0.383 | 0.020 | 4.261×10^-3^ | 115082 | 8.841×10^-5^ | 10.175 |
| rs74320788 | 0.161 | -0.025 | 5.643×10^-3^ | 115082 | 4.662×10^-5^ | 5.365 |
| rs1890896 | 0.527 | -0.019 | 4.149×10^-3^ | 115082 | 8.901×10^-5^ | 10.244 |
| rs140904868 | 0.576 | 0.020 | 4.342×10^-3^ | 115082 | 8.895×10^-5^ | 10.237 |
| rs11057837 | 0.101 | 0.030 | 6.878×10^-3^ | 115082 | 3.018×10^-5^ | 3.474 |
| rs11571787 | 0.214 | -0.024 | 5.041×10^-3^ | 115082 | 6.842×10^-5^ | 7.874 |
| rs41356552 | 0.203 | -0.024 | 5.141×10^-3^ | 115082 | 6.260×10^-5^ | 7.205 |
| rs79829764 | 0.060 | -0.040 | 9.020×10^-3^ | 115082 | 1.901×10^-5^ | 2.188 |
| rs111784051 | 0.030 | -0.423 | 1.213×10^-2^ | 115082 | 6.211×10^-4^ | 71.517 |
| rs79915079 | 0.026 | -0.086 | 1.301×10^-2^ | 115082 | 1.917×10^-5^ | 2.207 |
| rs2865505 | 0.310 | -0.027 | 4.484×10^-3^ | 115082 | 1.328×10^-4^ | 15.282 |
| rs6016505 | 0.550 | 0.027 | 4.144×10^-3^ | 115082 | 1.764×10^-4^ | 20.307 |
| rs6073958 | 0.199 | 0.064 | 5.192×10^-3^ | 115082 | 4.217×10^-4^ | 48.549 |
| rs6076364 | 0.414 | -0.019 | 4.197×10^-3^ | 115082 | 8.465×10^-5^ | 9.742 |

Abbreviations: EAF, effect allele frequency.

**Table S5.** SNPs information and F statistics of L-LDL-C.

| **RSID** | | **EAF** | **β** | **Standard error of β** | **Sample size** | | **R^2^** | | **F** | |
| --- | --- | --- | --- | --- | --- | --- | --- | --- | --- | --- |
| rs79414984 | | 0.021 | 0.064 | 1.442×10^-2^ | 115082 | | 1.224×10^-4^ | | 14.086 | |
| rs2642438 | | 0.704 | 0.025 | 4.472×10^-3^ | 115082 | | 7.075×10^-6^ | | 0.814 | |
| rs12472790 | | 0.496 | 0.019 | 4.090×10^-3^ | 115082 | | 1.141×10^-4^ | | 13.135 | |
| rs55709272 | | 0.437 | -0.019 | 4.126×10^-3^ | 115082 | | 8.963×10^-5^ | | 10.316 | |
| rs562338 | | 0.820 | 0.108 | 5.301×10^-3^ | 115082 | | 8.885×10^-5^ | | 10.225 | |
| rs140798831 | | 0.657 | -0.026 | 4.312×10^-3^ | 115082 | | 1.072×10^-3^ | | 123.526 | |
| rs11127048 | | 0.614 | -0.032 | 4.259×10^-3^ | 115082 | | 1.398×10^-4^ | | 16.091 | |
| rs2287622 | | 0.604 | -0.021 | 4.174×10^-3^ | 115082 | | 2.321×10^-4^ | | 26.712 | |
| rs9880155 | | 0.026 | -0.054 | 1.268×10^-2^ | 115082 | | 1.100×10^-4^ | | 12.656 | |
| rs62251256 | | 0.018 | -0.077 | 1.670×10^-2^ | 115082 | | 8.088×10^-6^ | | 0.931 | |
| rs34101748 | | 0.046 | -0.050 | 1.007×10^-2^ | 115082 | | 1.910×10^-5^ | | 2.198 | |
| rs2123886 | | 0.314 | 0.020 | 4.536×10^-3^ | 115082 | | 7.310×10^-5^ | | 8.413 | |
| rs2610992 | | 0.649 | -0.019 | 4.322×10^-3^ | 115082 | | 7.396×10^-5^ | | 8.512 | |
| rs59950280 | | 0.331 | 0.022 | 4.393×10^-3^ | 115082 | | 9.761×10^-5^ | | 11.234 | |
| rs116734477 | | 0.041 | -0.066 | 1.030×10^-2^ | 115082 | | 2.816×10^-5^ | | 3.241 | |
| rs3846662 | | 0.424 | 0.055 | 4.13×10^-3^ | 115082 | | 7.457×10^-4^ | | 85.875 | |
| rs36018387 | | 0.105 | -0.038 | 6.681×10^-3^ | 115082 | | 5.291×10^-5^ | | 6.090 | |
| rs12662901 | | 0.574 | -0.019 | 4.149×10^-3^ | 115082 | | 9.183×10^-5^ | | 10.569 | |
| rs1800769 | | 0.169 | -0.027 | 5.512×10^-3^ | 115082 | | 6.002×10^-5^ | | 6.907 | |
| rs2268702 | | 0.310 | 0.021 | 4.432×10^-3^ | 115082 | | 8.090×10^-5^ | | 9.311 | |
| rs147841808 | | 0.132 | 0.048 | 6.151×10^-3^ | 115082 | | 1.222×10^-4^ | | 14.061 | |
| rs1057558 | | 0.220 | -0.021 | 4.930×10^-3^ | 115082 | | 5.582×10^-5^ | | 6.424 | |
| rs147787064 | | 0.017 | -0.074 | 1.594×10^-2^ | 115082 | | 6.297×10^-5^ | | 0.725 | |
| rs4722551 | | 0.158 | 0.030 | 5.590×10^-3^ | 115082 | | 6.724×10^-5^ | | 7.738 | |
| rs73064442 | | 0.162 | 0.024 | 5.551×10^-3^ | 115082 | | 4.572×10^-5^ | | 5.261 | |
| rs74340693 | | 0.019 | -0.071 | 1.510×10^-2^ | 115082 | | 7.291×10^-6^ | | 0.839 | |
| rs17411113 | | 0.100 | 0.032 | 6.800×10^-3^ | 115082 | | 3.558×10^-5^ | | 4.0945 | |
| rs1461729 | | 0.899 | 0.063 | 6.791×10^-3^ | 115082 | | 1.368×10^-4^ | | 15.745 | |
| rs11789603 | | 0.109 | 0.035 | 6.572×10^-3^ | 115082 | | 4.898×10^-5^ | | 5.637 | |
| rs4008004 | | 0.222 | 0.025 | 4.936×10^-3^ | 115082 | | 7.480×10^-5^ | | 8.608 | |
| rs76265243 | | 0.035 | -0.051 | 1.147×10^-2^ | 115082 | | 1.151×10^-5^ | | 1.325 | |
| rs72805692 | | 0.114 | -0.031 | 6.421×10^-3^ | 115082 | | 4.116×10^-5^ | | 4.737 | |
| rs2792735 | | 0.720 | -0.023 | 4.549×10^-3^ | 115082 | | 8.824×10^-5^ | | 10.156 | |
| rs7918142 | | 0.457 | 0.019 | 4.144×10^-3^ | 115082 | | 9.094×10^-5^ | | 10.466 | |
| rs12246352 | | 0.103 | 0.030 | 6.741×10^-3^ | 115082 | | 3.245×10^-5^ | | 3.734 | |
| rs59379014 | | 0.073 | 0.057 | 7.847×10^-3^ | 115082 | | 6.153×10^-5^ | | 7.081 | |
| rs2229738 | | 0.066 | -0.040 | 8.195×10^-3^ | 115082 | | 2.508×10^-5^ | | 2.886 | |
| rs525028 | | 0.709 | -0.032 | 4.514×10^-3^ | 115082 | | 1.832×10^-4^ | | 21.087 | |
| rs11065358 | | 0.662 | -0.020 | 4.335×10^-3^ | 115082 | | 8.490×10^-5^ | | 9.771 | |
| rs4766237 | | 0.065 | -0.036 | 8.348×10^-3^ | 115082 | | 1.921×10^-5^ | | 2.210 | |
| rs7968813 | | 0.381 | -0.022 | 4.270×10^-3^ | 115082 | | 1.111×10^-4^ | | 12.782 | |
| **RSID** | **EAF** | **β** | **Standard error of β** | | **Sample size** | | **R^2^** | | **F** | |
| rs261290 | 0.655 | -0.041 | 4.305×10^-3^ | | 115082 | | 3.641×10^-4^ | | 41.920 | |
| rs633695 | 0.292 | 0.034 | 4.507×10^-3^ | | 115082 | | 2.052×10^-4^ | | 23.623 | |
| rs75003668 | 0.033 | 0.057 | 1.154×10^-2^ | | 115082 | | 1.361×10^-5^ | | 1.566 | |
| rs72836561 | 0.031 | -0.057 | 1.169×10^-2^ | | 115082 | | 1.262×10^-5^ | | 1.453 | |
| rs77960347 | 0.013 | 0.096 | 1.784×10^-2^ | | 115082 | | 6.525×10^-6^ | | 0.751 | |
| rs79120103 | 0.031 | -0.053 | 1.193×10^-2^ | | 115082 | | 1.054×10^-5^ | | 1.212 | |
| rs147711004 | 0.036 | 0.138 | 1.117×10^-2^ | | 115082 | | 9.161×10^-5^ | | 10.543 | |
| rs118170342 | 0.041 | 0.131 | 1.046×10^-2^ | | 115082 | | 1.064×10^-4^ | | 12.251 | |
| rs10423694 | 0.703 | -0.024 | 4.516×10^-3^ | | 115082 | | 9.871×10^-5^ | | 11.360 | |
| rs7254892 | 0.032 | -0.451 | 1.158×10^-2^ | | 115082 | | 8.152×10^-4^ | | 93.890 | |
| rs73024931 | 0.158 | -0.026 | 5.651×10^-3^ | | 115082 | | 4.966×10^-5^ | | 5.715 | |
| rs2569550 | 0.593 | 0.030 | 4.159×10^-3^ | | 115082 | | 2.130×10^-4^ | | 24.523 | |
| rs73147887 | 0.224 | 0.023 | 4.977×10^-3^ | | 115082 | | 6.443×10^-5^ | | 7.415 | |
| rs1800961 | 0.030 | -0.067 | 1.193×10^-2^ | | 115082 | | 1.594×10^-5^ | | 1.834 | |

Abbreviations: EAF, effect allele frequency.

**Table S6.** The number of total IVs and IVs with F-statistics below 10 in the univariable MR analysis.

| **Exposures** | **Outcomes** | **Numbers of total IVs** | **Numbers of IVs with F<10** |
| --- | --- | --- | --- |
| S-LDL-C | AIS | 25 | 15 |
| S-LDL-C | LAS | 23 | 14 |
| S-LDL-C | SVS | 21 | 11 |
| S-LDL-C | CES | 25 | 15 |
| M-LDL-C | AIS | 25 | 16 |
| M-LDL-C | LAS | 23 | 14 |
| M-LDL-C | SVS | 24 | 15 |
| M-LDL-C | CES | 25 | 16 |
| L-LDL-C | AIS | 55 | 32 |
| L-LDL-C | LAS | 51 | 29 |
| L-LDL-C | SVS | 50 | 29 |
| L-LDL-C | CES | 55 | 32 |

Abbreviations: IVs, instrumental variables; S-LDL-C, small low-density lipoprotein cholesterol; M-LDL-C, medium low-density lipoprotein cholesterol; L-LDL-C, large low-density lipoprotein cholesterol; AIS, any ischemic stroke; LAS, large artery stroke; SVS, small vessel stroke; CES, cardioembolic stroke.

**Table S7.** Results of I_GX_^2^ statistics in the univariable MR analysis.

| **Exposures** | **Outcomes** | **I_GX_^2^** |
| --- | --- | --- |
| S-LDL-C | AIS | 0.989 |
| S-LDL-C | LAS | 0.990 |
| S-LDL-C | SVS | 0.991 |
| S-LDL-C | CES | 0.989 |
| M-LDL-C | AIS | 0.987 |
| M-LDL-C | LAS | 0.988 |
| M-LDL-C | SVS | 0.987 |
| M-LDL-C | CES | 0.987 |
| L-LDL-C | AIS | 0.986 |
| L-LDL-C | LAS | 0.987 |
| L-LDL-C | SVS | 0.986 |
| L-LDL-C | CES | 0.986 |

Abbreviations: S-LDL-C, small low-density lipoprotein cholesterol; AIS, any ischemic stroke; LAS, large artery stroke; SVS, small vessel stroke; CES, cardioembolic stroke; M-LDL-C, medium low-density lipoprotein cholesterol; L-LDL-C, large low-density lipoprotein cholesterol.

**Figure S1:** Univariable MR estimates for the causal effect of total LDL-C on AIS, LAS, SVS and CES.


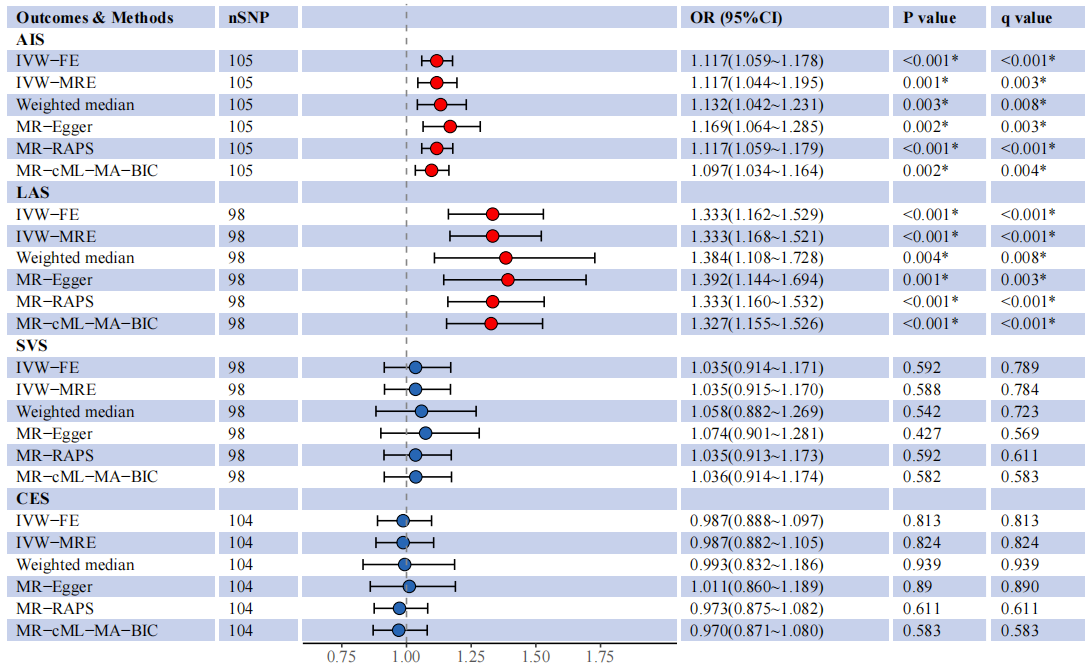


Abbreviations: SNP, single-nucleotide polymorphism; OR, odds ratio; CI, confidence interval; AIS, any ischemic stroke; IVW-FE, inverse variance weighted fixed effects; IVE-MRE, inverse variance weighted multiplicative random effects; MR-RAPS, MR-robust adjusted profile score; MR-cML-MA-BIC, MR-constrained maximum likelihood and model averaging and Bayesian information criterion; LAS, large artery stroke; SVS, small vessel stroke; CES, cardioembolic stroke.

**Figure S2:** Leave-one-out analysis for the causal effect of LDL-C on IS and its subtypes.


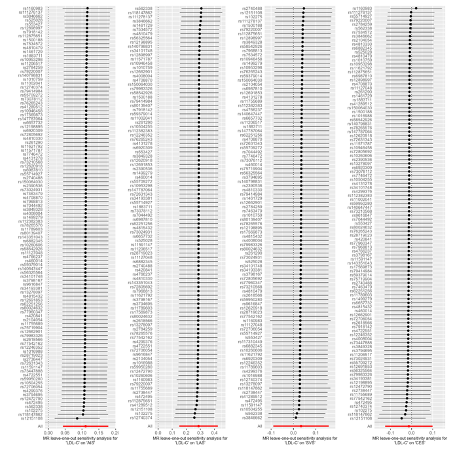


**Figure S3:** Leave-one-out analysis for the causal effect of S-LDL-C on IS and its subtypes.


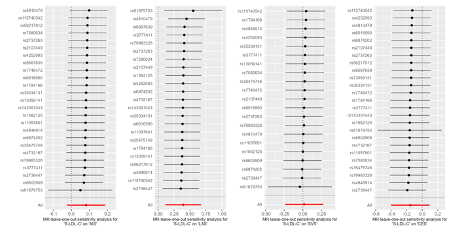


**Figure S4:** Leave-one-out analysis for the causal effect of M-LDL-C on IS and its subtypes.


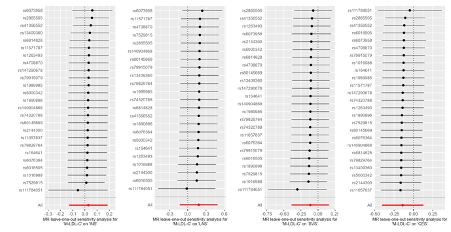


**Figure S5:** Leave-one-out analysis for the causal effect of L-LDL-C on IS and its subtypes.


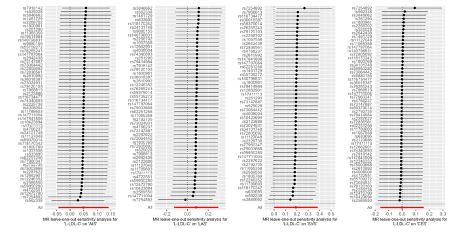

Supplement: Supplementary file 1 [file DataSheet_1.docx]
